# Using DNA Barcoding to Assess Caribbean Reef Fish Biodiversity: Expanding Taxonomic and Geographic Coverage

**DOI:** 10.1371/journal.pone.0041059

**Published:** 2012-07-17

**Authors:** Lee A. Weigt, Carole C. Baldwin, Amy Driskell, David G. Smith, Andrea Ormos, Eric A. Reyier

**Affiliations:** 1 Laboratories of Analytical Biology, National Museum of Natural History, Smithsonian Institution, Washington, D.C., United States of America; 2 Department of Vertebrate Zoology, National Museum of Natural History, Smithsonian Institution, Washington, D.C., United States of America; 3 Kennedy Space Center Ecological Program, IHA Environmental Services, Kennedy Space Center, Florida, United States of America; American Museum of Natural History, United States of America

## Abstract

This paper represents a DNA barcode data release for 3,400 specimens representing 521 species of fishes from 6 areas across the Caribbean and western central Atlantic regions (FAO Region 31). Merged with our prior published data, the combined efforts result in 3,964 specimens representing 572 species of marine fishes and constitute one of the most comprehensive DNA barcoding “coverages” for a region reported to date. The barcode data are providing new insights into Caribbean shorefish diversity, allowing for more and more accurate DNA-based identifications of larvae, juveniles, and unknown specimens. Examples are given correcting previous work that was erroneous due to database incompleteness.

## Introduction

The use and acceptance of DNA barcoding for animal identification has proliferated to many life science disciplines and other areas of human concern. Critical to the utility of the methods is the ability to put into context the DNA barcoding results, which are dependent on a database of all the possible sequence matches. In relatively simple studies [1] where there are relatively few potential targets (all terrestrial vertebrates in a region) a correct identification of unknowns is likely due to the relative ease of obtaining sequences of all possible matches. In complex systems (all the invertebrates in the ocean, all insects in a tropical terrestrial system), the absolute numbers of possible “answers” for the DNA barcode of an unknown specimen increases greatly, but the probability of making an identification decreases to a great degree because of the vast size of the database of all possible matches. This fact only increases the challenges to the portion of DNA barcoding that is the effort to build the reference library against which to query samples. For example, if there are four possible answers in a simple case, and the database contains two of them, then you have a 50% chance of a positive “hit” or match to the database. However, as the two other species are lacking, there is no confirmation that the barcodes from these two taxa are different from the barcodes of the two taxa present in the database. The same is true if there are 1,000 or 10,000 species in the system or area of exploration, and only half are present in the reference library. The complexity of data interpretation increases further when geographic sampling and any potential individual or population variation are taken into account.

Fish barcoding projects have been undertaken in many geographic regions [2]–[9] yet most are not yet approaching comprehensive taxonomic coverage, at least not for large, marine, or highly biodiverse regions. Additionally, specific taxonomic groups have been targeted regionally or globally [10]–[19], and though the entire enterprise is well planned and managed [20]–[22], rarely have the geographically comprehensive projects been broad in taxonomic coverage, similar to an all-taxon biotic inventory approach. In 2002 we designed and conducted a proof-of-concept effort using DNA sequences (four mitochondrial loci) to identify marine fish larvae to species, a task that is difficult using conventional morphological methods because the planktonic larvae of many marine fishes bear little resemblance to the adults they will become. By the end of 2003 we had sufficient data in hand from Caribbean collection efforts to incorporate molecular identifications into our standard larval fish (and fish egg) workflows, as have others [23]–[26]. As the data accumulated, it became apparent that scores of DNA sequences from fish larvae were not matching any sequences from catalogued adult specimens, and we shifted efforts to place a primary emphasis on collecting all the adults in each region. When complete, such a collection would allow researchers to far more effectively match unknown larval or other fish sequences and provide a much higher probability of a confident species-level identification because of the more comprehensive coverage of the database of “knowns”. In this study, we report on the dramatic increase in both the taxonomic and geographic DNA barcode sampling of fishes of the Caribbean and FAO region 31 due to our efforts, and show how this informs and increases identification success and sheds new light on species diversity in the Caribbean.

**Table 1:** Collection efforts (by BOLD project) resulting in specimens reported here.

BOLD Project	Country-State	Year	Samples reported
BAHA	Bahamas	2008	229
BZLWA	Belize	2004	476
BZLWB	Belize	2005	366
BZLWC	Belize	2006	293
BZLWD	Belize	2007	387
BZLWE	Belize	2008	415
CURA	Curaçao	2008	369
FCCA	USA-Florida	2009	139
SMSA	USA-Florida	2007	407
TOBA	Tobago	2009	343

**Figure 1:**
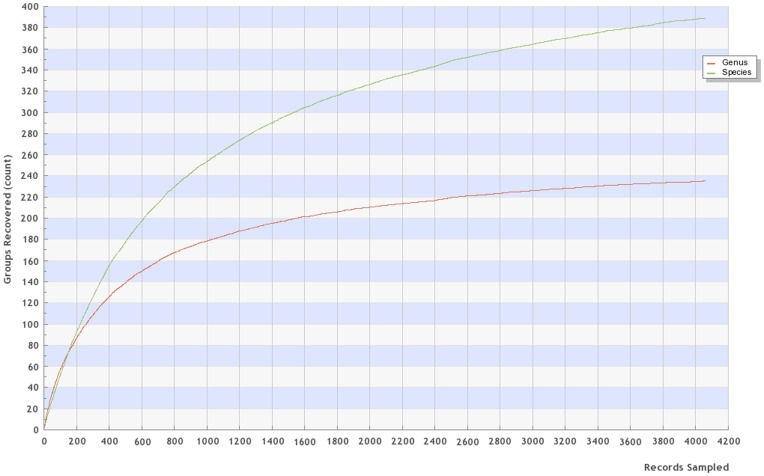
BOLD-generated accumulation curves for specimens in this study.

## Materials and Methods

Table 1 lists major collection efforts over six years in the countries by BOLD project name, country, number of samples successfully barcoded and reported here. Each trip represents many collection events. Additional samples from these collection efforts have been previously reported [10]–[14], [17].

**Table 2:** Coverage reported here of the speciose fish families listed by Floeter et al. [42] for the Western Atlantic (WA).

Family	WAgenera	generaherein	% genericcoverage
Apogonidae	3	3	100%
Balistidae	4	3	75%
Batrachoididae	5	4	80%
Blenniidae	7	6	86%
Carangidae	10	8	80%
Chaenopsidae	10	5	50%
Dactyloscopidae	6	3	50%
Gobiesocidae	6	4	67%
Gobiidae	25	18	72%
Haemulidae	3	3	100%
Labridae	8	7	88%
Labrisomidae	5	4	80%
Lutjanidae	6	4	67%
Muraenidae	8	5	63%
Pomacentridae	4	4	100%
Scaridae	4	3	75%
Serranidae	27	11	41%
Sparidae	6	4	67%
Syngnathidae	9	7	78%
Tripterygidae	1	1	100%
Total genera	158	107	68%

Fish specimens were collected with anesthetics, ichthyocides, cast nets, seines, benthic trawls, and pole spears, and a few were purchased from local fishermen. Upon collection and morphological sorting, fish were identified to the extent possible in the field, photographed (in the field whenever possible for best living color representation), and biopsied for tissues for subsequent molecular work. Many DNA extraction and PCR/DNA sequencing protocols have been published specific to special cases of preservation or tissues [27]–[33], but our methods across this project have not required special consideration other than generating high-quality DNA extractions for archival purposes [34]–[37]. Voucher specimens were preserved for permanent archival in the Smithsonian’s marine collections. Tissues and archival organic DNA extractions are submitted for permanent archival to the Smithsonian/NMNH Biorepository. Calculations of project statistics were done using the BOLD project workbench (www.boldsystems.org), the BOLI data portal tools (boli.uvm.edu), and the species delimitation plug-in for the Geneious software package [38]. Strictly for visualization purposes, neighbor-joining trees [39] were constructed using Kimura 2-parameter distances [40] utilizing BOLD, Paup* [41], and FigTree [42].

**Table 3:** Mexican larvae and juveniles with improved identifications (in boldface) due to increased taxon and geographic sampling. Original data from Valdez-Moreno et al [24], [25].

	Original Identification			Revised Identification		
ID #	Family	Genus	species	Family	Genus	species
MFL882	Carangidae	*Decapterus*		Carangidae	***Elagatis***	***bipinnulata***
MFL750	Haemulidae	*Haemulon*		Haemulidae	***Anisotremus***	***virginicus***
MFL1494	Kyphosidae	*Kyphosus*		Kyphosidae	*Kyphosus*	***incisor***
MX1339	Belonidae	*Strongylura*		Belonidae	*Strongylura*	***timicu***
MFL851	Scaridae	*Sparisoma*	*sp2*	Scaridae	*Sparisoma*	***atomarium***
MFL690	Tetraodontidae	*Sphoeroides*		Tetraodontidae	*Sphoeroides*	***nephelus***
MFL684	Ophidiidae			Ophidiidae	***Ogilbia***	
MFL0017	Labrisomidae			Labrisomidae	***Labrisomus***	
MFL796	Labrisomidae			Labrisomidae	***Malacoctenus***	
MFL0006	Labrisomidae			Labrisomidae	***Malacoctenus***	
MFL677	Clupeidae	*Jenkinsia*		Clupeidae	*Jenkinsia*	***lamprotaenia***
MFL830	Ophidiidae			Ophidiidae	***Parophidion***	***schmidti***
MFL856	Gobiidae			Gobiidae	***Gobionellus***	***oceanicus***
MFL855	Gobiidae	*Ctenogobius*	*boleosoma*	Gobiidae	*Ctenogobius*	***saepepallens***
MFL859	Gobiidae			Gobiidae	***Ctenogobius***	
MFL790	Microdesmidae			Microdesmidae	***Microdesmus***	***carri***
MFL683	Bothidae	*Bothus*	*ocellatus*	Bothidae	*Bothus*	***maculiferus***
MFL868	Balistidae	*Xanthichthys*	*ringens*	Balistidae	***Melichthys***	***niger***
MFL814	Tripterygiidae	*Enneanectes*		**Dactyloscopidae**	***Gillellus***	***uranidea***
MFL640	Scorpaenidae			Scorpaenidae	***Scorpaena***	***bergii***
MFL831	Scorpaenidae			Scorpaenidae	***Scorpaena***	***inermis***
MFL1488	Ostraciidae			Ostraciidae	***Lactophrys***	***trigonus***
MFL1516	Ophichthyidae			Ophichthyidae	***Myrichthys***	***ocellatus***
MFL863	Antennariidae			Antennariidae	***Antennarius***	***pauciradiatus***
MFL503	Gerreidae	*Eucinostomus*		Gerreidae	*Eucinostomus*	***harengulus***
MFL1510				**Sciaenidae**	***Pareques***	***umbrosus***

**Figure 2:**
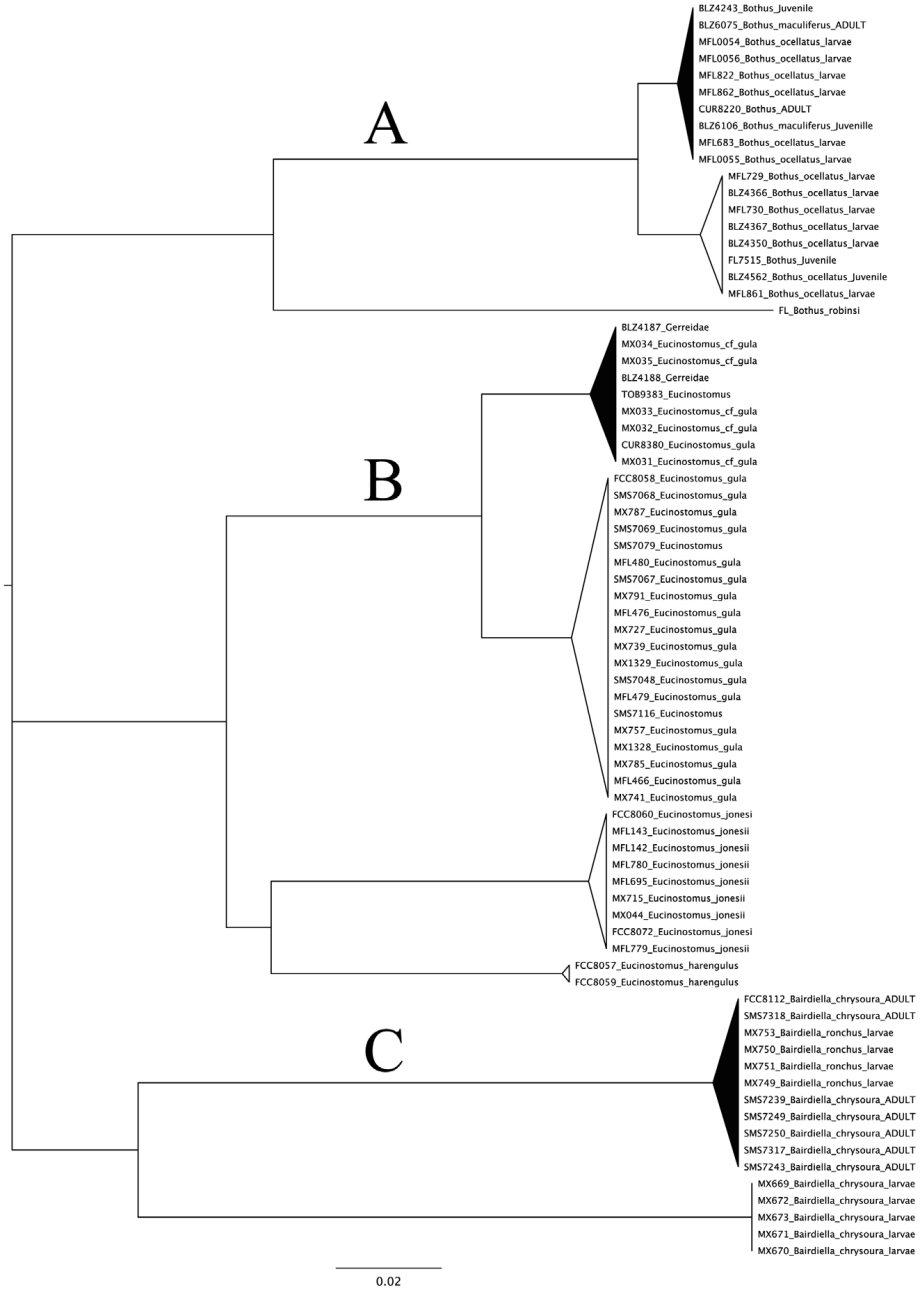
Dendrogram including three instances of specimen and taxonomic confusion discussed in the text. Combined barcoding data from Mexico (MFL- and MX- prefixes) [24], [25] with our data (BLZ = Belize; CUR = Curacao; FCC = Florida; FL = Florida; SMS = Florida) and shown in a dendrogram for visualization purposes only, with no intent to show phylogenetic relationships. The triangles are to scale showing the maximum amount of genetic variation within the clade, which ranges from the straight line (no differences between individuals) of the bottom clade (*Bairdiella chrysoura*) to the largest intraspecific variation in the *Eucinostomus gula* clade (B-white triangle: largest pairwise genetic distance = 0.0894) Interspecific and inter-clade distances and significance is discussed in the text.

## Results

All data are publicly available in BOLD public projects (listed in Table 1;www.boldsystems.org) and in GenBank (Accession numbers: JQ839691–JQ840387; JQ840390–JQ840744; JQ840747–JQ841039; JQ841042–JQ843096). Previously published projects now public on BOLD {APG (*Apogon*), BATHY (*Bathygobius*), CORY (*Coryphopterus*), PHAE (*Phaeoptyx* and *Astrapogon*), RYP (*Rypticus*), and STARK (*Starksia*)} are also comprised of specimens collected as part of this project but that were subjected to taxonomic evaluation prior to this evaluation. The data from those projects have been included in the statistical analyses (intraspecific and interspecific calculations) for the purposes of this report.

The accumulation curve (output from BOLD) of species, genera and “barcode clusters” is shown in Figure 1, and only includes specimens named to the species or genus level. For ease of re-evaluation and visualization, the nexus file of all specimens is provided in Table S1. Floeter et al. [43] provided a useful, though not comprehensive, metric utilizing 20 speciose families in the region [44], [45] that include 158 genera. Table 2 shows our data converging towards comprehensive generic coverage for those families. Our groups collecting efforts here provide an additional 16 genera from the region not previously reported (*Alectis, Arcos, Bathyanthias, Bollmannia, Bullisichthys, Coralliozetus, Decodon, Diplectrum, Gonioplectrus, Hemanthias, Hypsoblennius, Jeboehlkia, Pagrus, Pristipomoides, Pronotogrammus, Seriola*), and BOLD has specimens from an additional 15 genera (*Anthias, Chaenopsis, Chriolepis, Dermatolepis, Ekemblemaria, Etelis, Gobulus, Hemicaranx, Hemiemblemaria, Nicholsina, Pycnomma, Schultzea, Serraniculus, Stenotomus, Xanthichthys*). Genera currently lacking from these speciose families will then be subsequently reduced to: *Amphichthys, Anarchias, Anarchopterus, Channomuraena, Dactylagnus, Derilissus, Evermannichthys, Halicampus, Leurochilus, Myxodagnus, Muraena, Nemaclinus, Palatogobius, Paralabrax, Parasphyraenops, Parella, Pariah, Plectranthias, Protemblemaria, and Vomerogobius.*

## Discussion

Our team places a strong emphasis on resolving taxonomic issues resulting from DNA barcoding both to improve our understanding of Caribbean shorefish diversity and increase our ability to successfully identify larvae. Extensive taxonomic and specimen review has resulted in several publications prior to this release of shallow water Caribbean fish data [10]–[14], [17]. Combined with the data released here, DNA barcode data for more than half of the 1023 species in the Western Atlantic are now available [43]. Occasionally, issues arise because DNA barcoding indicates the potential presence of more lineages in a geographic location than are currently recognized [10] and nine new species have already been described [10], [12], in addition to numerous others awaiting description. Rather than further delaying the release of the data presented herein pending additional revisions and species descriptions, we withhold species names of certain specimens (513 specimens or 15% of the total). Genera that fall into this category include: *Acanthemblemaria*, *Acyrtops*, *Acyrtus*, *Elacatinus*, *Enneanectes*, *Gobiesox*, *Lythrypnus*, *Malacoctenus*, *Odontoscion, Pareques, Petrotyx, Platygillellus, Priolepis*, *Risor*, and *Synodus*. Remaining specimens without associated species names are due to difficulties identifying specimens confidently to species. There are two distinct issues limiting species-level identification: 1) barcoding (of early life history stages in particular; 683 specimens in this data set, 20% of total) suggests that the adult form has not yet been collected and identified; and 2) specimens in-hand cannot yet be unambiguously assigned to a single species because of extreme morphological similarities (315 specimens, 9.3% of total). Rather than introducing more confusion by potentially incorrectly applying species names, we have chosen to leave the identifications at family or genus and allow for subsequent further investigation by us or other taxonomic experts. We continue to strive for data release earlier rather than later and therefore publish the data with the specimens identified only to the level to which there is currently high confidence. This has the added advantage of not resulting in matches to sequences in the database that are misidentified, which can cause confusion. One of the strengths of the BARCODE “flag” in GenBank is the confidence that much effort has been placed on not perpetuating incorrect information, much confidence can be placed on the identification of the specimen that was sequenced, and should a question arise in terms of identification, the location of the voucher specimen in an accessible collection has been documented.

Ward et al. [8] found the average interspecific variation in 207 species of Australian fishes to be 9.93%, and Hubert et al. [9] found an average 8.30% interspecific distance for 193 species of Canadian freshwater fishes. For animal species in general, Kartavtsev [46], [47] reported an average intergeneric distance of 16.6%. Our data (all in BOLD and calculated using BOLD analytical tools) show and average interspecific (intra-generic) distance of 16.3% (+0.026%). Calculations of averages distances to the “nearest-neighbor”, instead of just inter-specific distances, average 11.95% (+0.02%). If taxa not identified to the species level are removed, then interspecific and nearest neighbor distances average 16.11% (+0.026) and 12.08% (+0.02) respectively.

Earlier efforts [48], [49] to identify Caribbean fish larvae without the aid of DNA were only marginally successful. Larvae have led to discoveries and descriptions of new species of fish including confirmed with molecular data [50], though not always with the COI barcode region. Through the use of DNA barcode data, we have realized an increase in the number of larval fishes identified to species from about 50 in 2004 to over 168 currently. Resolution of taxonomic issues highlighted by barcode data (e.g., [17]) also has allowed identification of larvae that were previously misidentified due to poorly resolved species classifications. By increasing the coverage of species and numbers of individuals through broader and repeated sampling, greater accuracy of identification of unknowns has now been enabled.

Valdez-Moreno et al. [24], [25] also studied fish in the western Caribbean testing some adults, but a majority of samples were larvae and eggs that they attempted to identify by matching the barcode sequences to known BOLD records. Their sampling included 181 species, 136 genera and 74 families. Of their 782 larval and egg specimens, 137 failed to match any records and 75 barcode lineages failed to have a close correspondence with existing records. By increasing taxonomic and geographic coverage, we have shed more light on these identifications and have found several incorrect identifications, as well as some taxonomic issues. Table 3 lists the corrected identifications of some of the larval records reported [24], [25].

Combining the barcoding data from Mexico [24], [25] with ours proved informative in other ways as well. Three interesting cases arise which merit further discussion and are shown in a dendrogram for visualization purposes only in Figure 2, with no intent to show phylogenetic relationships. The first case (A in Figure 2) is of taxa that are similar enough to fall in the range of differentiation that can be due to either closely related species or genetically more divergent populations of the same species. This occurs in the flatfish genus *Bothus*, and the samples from the Mexican studies [24], [25] “MFL” in Fig. 2) are identified as conspecifics, fall into two groups and have Kimura 2-parameter genetic distances averaging 1.30% (range = 0.00–2.55%). All of these MFL and MX samples are larvae. This would be within the expected range of intraspecific variation for species maintaining a broad geographic range, but elevated for specimens collected in close proximity [8], [51]. Do these specimens represent the high end of the range of intraspecific variation, or the low end of the range of interspecific variation? Without full taxonomic coverage, it can be difficult to determine, as both are realistic possibilities. However, when put into context with nearest neighbor taxa and other congeners via more comprehensive taxon sampling, the two larvae in question fall into reciprocally monophyletic groups, each with the appropriate species identification based on juveniles or adults. The resulting calculations of intraspecific divergences would be very low with *Bothus maculiferus* at 0.12% (range 0.00%–0.31%) and *Bothus ocellatus* at 0.31% (range 0.00%–0.62%) and the interspecific divergences of 2.31% (range 2.04%–2.55%) showing a sizeable “barcode gap” over the intraspecific values.

Problems and confusion with identifications of *Bothus* using the BOLD database will persist until the Mexican larval specimens are renamed (not necessarily to the other species name, but merely to genus). Once these specimens are renamed in BOLD and GenBank they will not continue to perpetuate problems and misidentifications. The specimens in question should not necessarily be renamed solely based on their DNA matching an adult from a different taxon, but due to the fact that the specimens in question are larvae (juveniles might also apply) and the possibility or potential of incorrect identification is high, or conversely, the confidence in the species name is low, while the confidence in the genus name remains high. Removing the species name and naming them *Bothus* in the database will eliminate these concerns, as well as correct the implied assertion that the barcoding method fails in this group, or doesn’t correctly distinguish between conspecifics, when it actually works very well.

The second case (B in Fig. 2) is part of a notoriously difficult to identify taxonomic group, the mojarras, specifically the genus *Eucinostomus*, which presents different problems in fresh versus preserved specimens. We have enlisted the assistance of a taxonomic expert to help us resolve this group, and will enlarge upon this study in the future. Specimens of *Eucinostomus gula* from the Atlantic coast of Florida and Mexico form the bottom group (clear triangle), whereas specimens from nearby Belize, Tobago and Curaçao form the other group (black triangle). But both groups also include specimens from Mexico. Were the Mexico specimens not present in both groups, we might not know if the observed level of sequence divergence indicated species level differences or geographic isolation patterns, but the occurrence in Mexico of specimens from both groups indicates significant isolation of gene flow between the two groups of mojarras.

The third case (C in Fig. 2) is another difficult to resolve scenario – this time in the sciaenid genus *Bairdiella*. Two closely related and difficult to identify taxa occur across the entire western Central Atlantic, *Bairdiella ronchus* and *B. chrysoura* [51]. The two taxa are have distinct geographic distributions: all Yucatan specimens (MX) are identified as *B. ronchus*, which is distributed throughout the Caribbean; while our Florida specimens (FCC and SMS) are identified as *B. chrysoura*, which has a more northern distribution including the east coast of the U.S. and northern Gulf of Mexico. Our Florida specimens (juveniles size range 17–36 mm; adults 85–130 mm) are from an area (east coast of Florida) only known to host one of the two taxa as adults and these key out to the lone species reported from the region, *Bairdiella chrysoura*. The larval specimens from Mexico are identified as two different species [24], [25], [51] but the two types appear to not show any differentiation. Adults of *Bairdiella ronchus* might shed some light on this dilemma, but until such specimens are acquired, it is apparent that we have some difficulty identifying larval *Bairdiella*, and it would be prudent to remove the species-level designations from larvae of this group. If the removal of the species-level designations for those larval specimens were to happen, there would be no internal conflict in the data for this genus. So frequently have these misrepresentations appeared in our datasets and those of others [52] that our group now takes a very conservative approach to placing names on specimens that cannot be reliably keyed out. This has the adverse effect of increasing the number of specimens identified only to genus (44.44% of specimens in this data release), but ultimately makes for a more robust overall database.

While improvements in identifications, corrections of misidentifications, and illumination of additional taxonomic issues that need resolution are not unexpected, it is still valuable to witness and document the increasing confidence in interpretation of results as we grow our datasets to levels of completion that will be of value to ecologists, taxonomists, and the scientific consumers of our biodiversity data worldwide.

## Supporting Information

Table S1 Aligned fasta-format file of all specimens. “Missing” sequence data from beginning and end of any sequences has been filled with “N’s” to avoid generating any alignment discrepancies. Title line for each specimen indicates Field Identification number and taxonomic identification.
(TXT)

## References

[1] Smith KM, Anthony SJ, Switzer WM, Epstein JH, Seimon T (2012). Zoonotic Viruses Associated with Illegally Imported Wildlife Products.. PLoS ONE.

[2] Aquilino SV, Tango JM, Fontanilla IKC, Pagulayan RC, Basiao ZU (2011). DNA Barcoding the ichthyofauna of Taal Lake, Philippines.. Molecular Ecology Resources.

[3] Ardura A, Linde AR, Moreira JC, Garcia-Vasquez E (2010). DNA barcoding for conservation and management of Amazonian commercial fish.. Biological Conservation.

[4] Asgharian H, Sahafi HH, Ardalan AA, Shekarriz S, Elahi E (2011). Cytochrome c oxidase subunit 1 barcode data of fish of the Nayband National Park in the Persian Gulf and analysis using meta-data flag several cryptic species.. Molecular Ecology Resources.

[5] Cawthorn D-M, HA Steinman, RC Witthuhn (2011). Establishment of a mitochondrial DNA sequence database for the identification of fish species commercially available in South Africa.. Molecular Ecology Resources.

[6] Lakra WS, MS Verma, M Goswami, KK Lal, V Mohindra (2011). DNA Barcoding Indian Marine Fishes.. Molecular Ecology Resources.

[7] Mecklenburg CW, Moller PR, Steinke D (2011). Biodiversity of arctic marine fishes: Taxonomy and zoogeography.. Marine Biodiversity.

[8] Ward RD, Zemlak TS, Innes BH, Last PR, Hebert PDN (2005). DNA barcoding Australia’s fish species.. Phil Trans Royal Soc B Biol Sci.

[9] Hubert N, Hanner R, Holm E, Mandrak NE, Taylor E (2008). Identifying Canadian freshwater fishes through DNA barcodes. PLoS One..

[10] Baldwin CC, Weigt LA (2012). A new species of soapfish (Teleostei: Serranidae: *Rypticus*), with redescription of *R. subbifrenatus* and comments on the use of DNA barcoding in systematic studies.. Copeia.

[11] Baldwin CC, Mounts JH, Smith DG, Weigt LA (2008). Genetic identification and color descriptions of early life-history stages of Belizean *Phaeoptyx* and *Astrapogon* (Teleostei: Apogonidae) with comments on identification of adult *Phaeoptyx*.. Zootaxa.

[12] Baldwin CC, Weigt LA, Smith DG, Mounts JH (2009). Reconciling genetic lineages with species in Western Atlantic *Coryphopterus* (Teleostei: Gobiidae).. Smithsonian Contributions to the Marine Sciences.

[13] Baldwin CC, Castillo CI, Weigt LA, Victor BC (2011). Seven new species within western Atlantic *Starksia atlantica*, *S. lepicoelia*, and *S. sluiteri* (Teleostei: Labrisomidae), with comments on congruence of DNA barcodes and species.. Zookeys.

[14] Baldwin CC, Brito BJ, Smith DG, Weigt LA, Escobar-Briones E (2011). Identification of early life-history stages of Caribbean *Apogon* species (Perciformes: Apogonidae) through DNA barcoding.. Zootaxa.

[15] Holmes BH, Steinke D, Ward RD (2009). Identification of shark and fin rays using DNA barcoding.. Fisheries Research.

[16] Hubert N, Delrieu-Trottin E, Irisson J-O, Meyer C, Planes S (2010). Identifying coral reef fish larvae through DNA barcoding: A test case with the families Acanthuridae and Holocentridae.. Molecular Phylogenetics and Evolution.

[17] Tornabene L, Baldwin C, Weigt LA, Pezold F (2010). Exploring the diversity of western Atlantic *Bathygobius* (Teleostei: Gobiidae) with cytochrome c oxidase-I, with descriptions of two new species.. Aqua.

[18] Wong EHK, Shivji MS, Hanner RH (2009). Identifying sharks with DNA barcodes: assessing the utility of a nucleotide diagnostic approach. Molecular Ecology Resources..

[19] Wang ZD, Guo YS, Tan W, Li L, Tang EP (2010). DNA barcoding, phylogenetic relationships and speciation of snappers (genus *Lutjanus*).. Science China Life Sciences.

[20] Becker S, Hanner R, Steinke D (2011). Five years of FISH-BOL: Brief status report.. Mitochondrial DNA.

[21] Ratnasingham S, Hebert P (2007). Molecular Ecology Notes. http://www.barcodinglife.org.

[22] Ward RD, Hanner R, Hebert PDN (2009). The campaign to DNA barcode all fishes, FISH-BOL.. Journal of Fisheries Biology.

[23] Aranishi F (2006). Single fish egg DNA extraction for PCR amplification.. Conservation Genetics.

[24] Valdez-Moreno M, Ivanova NV, Elias-Gutierrez M, Contreras-Balderas S, Hebert PDN (2009). Probing diversity in freshwater fishes from Mexico and Guatemala with DNA barcodes.. Journal of Fisheries Biology.

[25] Valdez-Moreno M, Vasquez-Yeomans L, Elias-Gutierrez M, Ivanova NV, Hebert PDN (2010). Using DNA barcodes to connect adults and early life stages of marine fishes from the Yucatan Peninsula, Mexico: potential in fisheries management.. Marine and Freshwater Research.

[26] Victor BC (2007). *Coryphopterus kuna*, a new goby (Perciformes: Gobiidae: Gobiinae) from the western Caribbean, with the identification of the late larval stage and an estimate of the pelagic larval duration.. Zootaxa.

[27] Bartlett SE, Davidson WS (1992). FINS (Forensically Informative Nucleotide Sequencing): a procedure for identifying the animal origin of biological specimens.. Biotechniques.

[28] Ivanova N, Zemlak TS, Hanner RH, Hebert PDN (2007). Universal primer cocktails for fish DNA barcoding.. Molecular Ecology Notes.

[29] Kumar R, Singh PJ, Nagpure NS, Kushwaha B, Srivastava SK (2007). A non-invasive technique for rapid extraction of DNA from fish scales.. Indian Journal of Experimental Biology.

[30] Lucentini L, Caporali S, Palomba A, Lancioni H, Panara F (2006). A comparison of conservative DNA extraction methods from fins and scales of freshwater fish: A useful tool for conservation genetics.. Conservation Genetics.

[31] Palero F, Hall S, Clark PF, Johnston D, Mackenzie-Dodds J (2010). DNA extraction from formalin-fixed tissue: new light from the deep sea.. Scientia Marina.

[32] Shokralla S, Singer GAC, Hajibabaei M (2010). Direct PCR amplification and sequencing of specimens’ DNA from preservative ethanol.. Biotechniques.

[33] Zhang J (2010). Exploiting formalin-preserved fish specimens for resources of DNA barcoding.. Molecular Ecology Resources.

[34] Handy SM, Deeds JR, Ivanova NV, Hebert PDN, Hanner R (2010). A single laboratory validated method for the generation of DNA Barcodes for the identification of fish for regulatory compliance.. Journal of AOAC International.

[35] Weigt LA, Driskell A, Ormos A, Meyer C, Collins A, DNA Barcodes: Methods, Protocols Kress, WJ, DL Erickson (2011). Introduction to animal barcoding protocols..

[36] Weigt LA, Driskell A, Ormos A, DNA Barcodes: Methods, Protocols Kress, WJ, DL Erickson (2011). DNA barcoding fishes..

[37] Yancy HF, Fry FS, Randolph SC, Deeds J, Ivanova NV (2008). A protocol for validation of DNA-barcoding for the species identification of fish for FDA regulatory compliance. FDA Laboratory Information Bulletin.. LIB No.

[38] Masters BC, Fan V, Ross HA (2011). Species delimitation – a geneious plugin for the exploration of species boundaries.. Molecular Ecology Resources.

[39] Saitou N, Nei M (1987). The neighbor-joining method: a new method for reconstructing phylogenetic trees.. Molecular Biology and Evolution.

[40] Kimura M (1980). A simple method for estimating evolutionary rates of base substitutions through comparative studies of nucleotide sequences.. Journal of Molecular Evolution.

[41] Swofford D (2002). Phylogenetic analysis using parsimony (* and other methods).. Sinauer Associates, Sunderland, Massachusetts.

[42] Figtree website.. http://tree.bio.ed.ac.uk/software/figtree/Accessed.

[43] Floeter SR, Rocha LA, Robertson DR, Joyeux JC, Smith-Vaniz WF (2008). Atlantic reef fish biogeography and evolution.. Journal of Biogeography.

[44] Eschmeyer WN (2010). Catalog of fishes website.. http://research.calacademy.org/research/ichthyology/catalog/speciesbyfamily.asp.

[45] Eschmeyer WN, Fricke R, Fong JD, Polack DA (2010). Marine fish diversity: history of knowledge and discovery (Pisces).. Zootaxa.

[46] Kartavtsev YP (2011). Sequence divergence at mitochondrial genes in animals: Applicability of DNA data in genetics of speciation and molecular phylogenetics.. Marine Genomics vol. 4 (2) 71–81.

[47] Kartavtsev YP (2011). Divergence at *Cyt-b* and *Co-1* mtDNA genes on different taxonomic levels and genetics of speciation in animals.. Mitochondrial DNA.

[48] Baldwin CC, Smith DG (2003). Larval Gobiidae (Teleostei: Perciformes) of Carrie Bow Cay, Belize, Central America.. *Bulletin of Marine Science*, 72, 639–674.

[49] Richards WJ (2006). Early stages of Atlantic fishes: an identification guide for western central North Atlantic.. CRC Press, Boca Raton, 2640 p.

[50] McBride RS, Rocha CR, Ruiz-Carus R, Bowen BW (2010). A new species of ladyfish, of the genus *Elops* (Elopiformes: Elopidae), from the western Atlantic Ocean.. Zootaxa.

[51] Ward RD, Holmes BH (2007). An analysis of nucleotide and amino acid variability in the barcode region of cytochrome c oxidase I (cox1) in fishes.. Molecular Ecology Notes.

[52] Chao NL (2002). Sciaenidae, 1601–1602.. In: The living marine resources of the Western Central Atlantic. Volume 3: Bony fishes part 2 (Opistognathidae to Molidae). Carpenter KE (Ed.). FAO species identification guide for fishery purposes and American Society of Ichthyologists and Herpetologists special publication no. 5. FAO, Rome..

[53] Vilgalys R (2003). Taxonomic misidentification in public DNA databases.. New Phytologist.

